# Depth Errors Analysis and Correction for Time-of-Flight (ToF) Cameras

**DOI:** 10.3390/s17010092

**Published:** 2017-01-05

**Authors:** Ying He, Bin Liang, Yu Zou, Jin He, Jun Yang

**Affiliations:** 1 Shenzhen Graduate School, Harbin Institute of Technology, Shenzhen 518055, China; bliang@tsinghua.edu.cn; 2 Department of Automation, Tsinghua University, Beijing 100084, China; y-zou10@mails.tsinghua.edu.cn (Y.Z.); he-j15@mails.tsinghua.edu.cn (J.H.); 3 Shenzhen Graduate School, Tsinghua University, Shenzhen 518055, China; yangjun603@mails.tsinghua.edu.cn

**Keywords:** ToF camera, depth error, error modeling, error correction, particle filter, SVM

## Abstract

Time-of-Flight (ToF) cameras, a technology which has developed rapidly in recent years, are 3D imaging sensors providing a depth image as well as an amplitude image with a high frame rate. As a ToF camera is limited by the imaging conditions and external environment, its captured data are always subject to certain errors. This paper analyzes the influence of typical external distractions including material, color, distance, lighting, etc. on the depth error of ToF cameras. Our experiments indicated that factors such as lighting, color, material, and distance could cause different influences on the depth error of ToF cameras. However, since the forms of errors are uncertain, it’s difficult to summarize them in a unified law. To further improve the measurement accuracy, this paper proposes an error correction method based on Particle Filter-Support Vector Machine (PF-SVM). Moreover, the experiment results showed that this method can effectively reduce the depth error of ToF cameras to 4.6 mm within its full measurement range (0.5–5 m).

## 1. Introduction

ToF cameras, which have been developed rapidly in recent years, are a kind of 3D imaging sensor providing a depth image as well as an amplitude image with a high frame rate. With its advantages of small size, light weight, compact structure and low power consumption, this equipment has shown great application potential in fields such as navigation of ground robots [1], pose estimation [2], 3D object reconstruction [3], identification and tracking of human organs [4] and so on. However, limited by its imaging conditions and influenced by the interference of the external environment, the data acquired by a ToF camera has certain errors, among which is the fact it has no unified correction method for any non-systematic errors caused by the external environment. Therefore, different depth errors must be analyzed, modeled and corrected case by case according to the different causes.

ToF camera errors can be divided into two categories: systematic errors, and non-systematic errors. A systematic error is triggered not only by its intrinsic properties, but also by the imaging conditions of the camera system. The main characteristic of this kind of error is that their form is relatively fixed. These errors can be evaluated in advance, and the correction process is relatively convenient. Systematic errors which can be reduced by calibration under normal circumstances [5] and can be divided into five categories.

A non-systematic error is an error caused by the external environment and noise. The characteristic of this kind of error is that the form is not fixed and random, and it is difficult to establish a unified model to describe and correct such errors. Non-systematic errors are mainly divided into four categories: signal-to-noise ratio, multiple light reception, light scattering and motion blurring [5].

Signal-to-noise ratio errors can be removed by the low amplitude filtering method [6], or an optimized integration time can be decided by using a complex algorithm as per the area to be optimized [7]. Other ways generally reduce the impact of noise by calculating the average of data to determine whether it exceeds a fixed threshold [8,9,10].

Multiple light reception errors mainly exist at surface edges and depressions of the target object. Usually, the errors in surface edges of the target object can be removed by comparing the incidence angle of the adjacent pixels [7,11,12], but there is no efficient solution to remove the errors of depressions in the target object.

Light scattering errors are only related to the position of a target object in the scene; the closer it is to the target object, the stronger the interference will be [13]. In [14], a filter approach based on amplitude and intensity on the basis of choosing an optimum integration time was proposed. Measurements based on multiple frequencies [15,16] and the ToF encoding method [17] both belong to the modeling category, which can solve the impact of sparse scattering. A direct light and global separation method [18] can solve mutual scattering and sub-surface scattering among the target objects.

In [19], the authors proposed detecting transverse moving objects by the combination of a color camera and a ToF camera. In [20], transverse and axial motion blurring were solved by an optical flow method and axial motion estimation. In [21], the authors proposed a fuzzy detection method by using a charge quantity relation so as to eliminate motion blurring.

In addition, some error correction methods cannot distinguish among error types, and uniformly correct the depth errors of ToF cameras. In order to correct the depth error of ToF cameras, a fusion method with a ToF camera and a color camera was also proposed in [22,23]. In [24], a 3D depth frame interpolation and interpolative temporal filtering method was proposed to increase the accuracy of ToF cameras.

Focusing on the non-systematic errors of ToF cameras, this paper starts with the analysis of the impacts of varying external distractions on the depth errors of ToF cameras, such as materials, colors, distances, and lighting. Moreover, based on the particle filter to select the parameters of a SVM error model, an error modeling method based on PF-SVM is proposed, and the depth error correction of ToF cameras is realized as well.

The reminder of the paper is organized as follows: Section 2 introduces the principle and development of ToF cameras. Section 3 analyzes the influence of lighting, material properties, color and distance on the depth errors of ToF cameras through four groups of experiments. In Section 4, a PF-SVM method is adopted to model and correct the depth errors. In Section 5, we present our conclusions and discuss possible future work.

## 2. Development and Principle of ToF Cameras

In a broad sense, ToF technology is a general term for determining distance by measuring the flight time of light between sensors and the target object surface. According to the different measurement methods of flight time, ToF technology can be classified into pulse/flash, continuous wave, pseudo-random number and compressed sensing [25]. The continuous wave flight time system is also called ToF camera.

**Figure 1:**
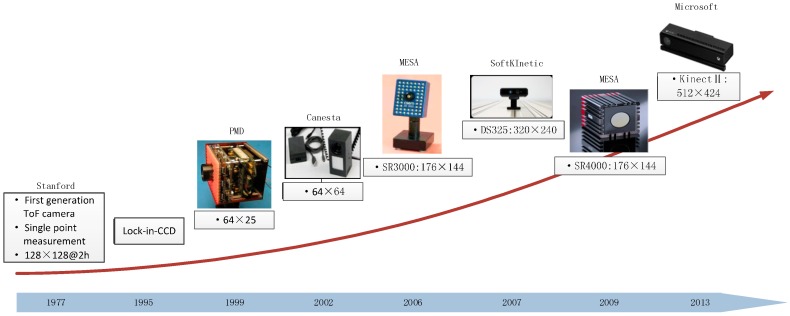
Development history of ToF cameras.

ToF cameras were firstly invented at the Stanford Research Institute (SRI) in 1977 [26]. Limited by the detector technology at that time, the technique wasn’t used widely. Fast sampling of receiving light didn’t come true until the lock-in CCD technique was invented in the 1990s [27]. Then, in 1997 Schwarte, who was at the University of Siegen (Germany), put forward a method of measuring the phases and/or magnitudes of electromagnetic waves based on the lock-in CCD technique [28]. With this technique, his team invented the first CCD-based ToF camera prototype [29]. Afterwards, ToF cameras began to develop rapidly. A brief development history is shown in Figure 1.

**Figure 2:**
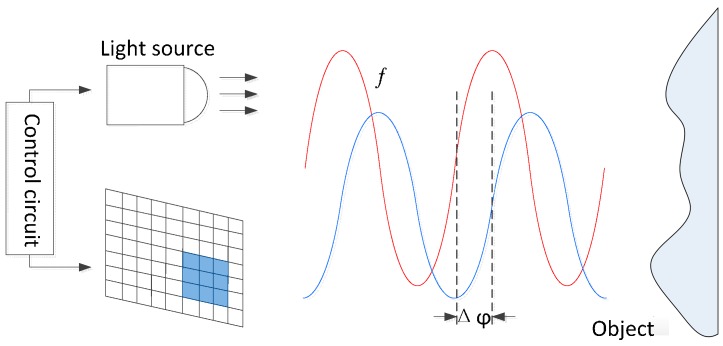
Principle of ToF cameras.

In Figure 2, the working principle of ToF cameras is illustrated. The signal is modulated on the light source (usually LED) and emitted to the surface of the target object. Then, the phase shift between the emitted and received signals is calculated by measuring the accumulated charge numbers of each pixel on the sensor. Thereby, we can obtain the distance from the ToF camera to the target object.

The received signal is sampled four times at equal intervals for every period (at 1/4 period). From the four samples (*ϕ*_0_, *ϕ*_1_, *ϕ*_2_, *ϕ*_3_) of phase *ϕ*, offset *B* and amplitude *A* can be calculated as follows:

(1) $$\varphi =\arctan\left(\frac{\varphi_{0}-\varphi_{2}}{\varphi_{1}-\varphi_{3}}\right),$$

(2) $$B=\frac{\varphi_{0}+\varphi_{1}+\varphi_{2}+\varphi_{3}}{4}$$

(3) $$A=\frac{\sqrt{{\left(\varphi_{0}-\varphi_{2}\right)}^{2}+{\left(\varphi_{1}-\varphi_{3}\right)}^{2}}}{2}$$

Distance *D* can be derived:

(4) $$D=\frac{1}{2}\left(\frac{c\Delta \varphi }{2\pi f}\right),$$

where *D* is the distance from ToF camera to the target object, *c* is light speed and *f* is the modulation frequency of the signal, Δφ is phase difference. More details on the principle of ToF cameras can be found in [5].

We list the exterior and parameters of several typical commercial ToF cameras on the market in Table 1.

## 3. Analysis on Depth Errors of ToF Cameras

The external environment usually has a random and uncertain influence on ToF cameras, therefore, it’s difficult to establish a unified model to describe and correct such errors. In this section, we take the MESA SR4000 camera (Zurich, Switzerland, a camera with good performance [30], which has been used in error analysis [31,32,33] and position estimation [34,35,36]) as an example to analyze the influence of the external environment transformation on the depth error of ToF cameras. The data we get from the experiments provide references for the correction of depth errors in the next step.

### 3.1. Influence of Lighting, Color and Distance on Depth Errors

During the measurement process of ToF cameras, it seems that the measured objects tend to have different colors, different distances and may be under different lighting conditions. Then, the following question arises: will the difference in lighting, distances and colors affect the measurement results? To answer this question, we conduct the following experiments.

As we know, there are several natural indoor lighting conditions, such as light-sunlight, indoor light-lamp light and no light. This experiment mainly considers the influence of these three lighting conditions on the depth errors of the SR4000. Red, green and blue are three primary colors that can be superimposed into any color. White is the color for measuring error [32,37,38], while reflective papers (tin foil) can reflect all light. Therefore, this experiment mainly considers the influence of these five conditions on the depth errors of the SR4000.

**Figure 3:**
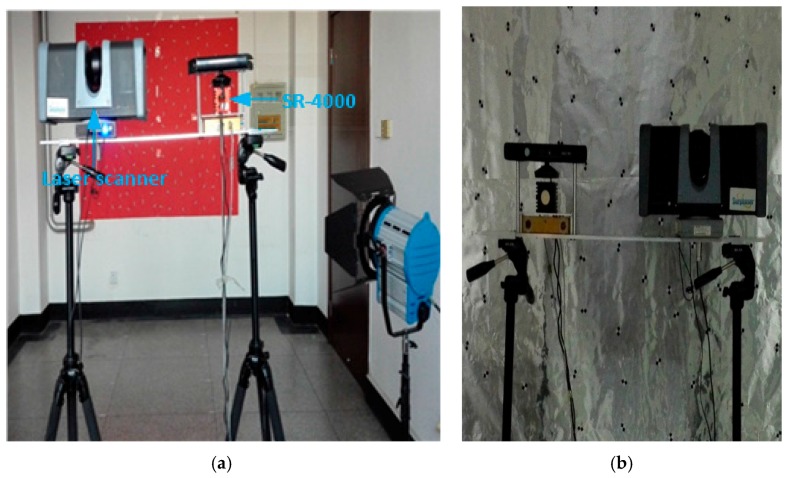
Experimental scene. (**a**) Experimental scene; (**b**) Camera bracket.

As the measurment target, the white wall is then covered by red, blue, green, white and reflective papers, respectively, as examples of backgrounds with different colors. Since the wall is not completely flat, laser scanners are used to build a wall model. Then we used a 25HSX laser scanner from Surphaser (Redmond, WA, USA) to provide a reference value, because its accuracy is relatively high (0.3 mm). The SR4000 camera is set on the right side of the bracket, while the 3D laser scanner is on the left. The bracket is mounted in the middle of two tripods and the tripods are placed parallel to the white wall. The distances between the tripods and the wall are measured with two parallel tapes. The experimental scene is arranged as shown in Figure 3 below.

The distances from the tripods to the wall are set to 5, 4, 3, 2.5, 2, 1.5, 1, 0.5 m respectively. At each position, we change the lighting conditions and obtain one frame with the laser scanner and 30 frames with the SR4000 camera. To exclude the influence of the integral time, the SR_3D_View software of the SR4000 camera is set to “Auto”.

In order to analyze the depth error, the acquired data are processed in MATLAB. Since the target object can’t fill the image, we select the central region of 90 × 90 pixels of the SR4000 to be analyzed for depth errors. The distance error is defined as:

(5) $$h_{i,j}=\frac{\sum_{f=1}^{n}m_{i,j,f}}{n}-r_{i,j},$$

(6) $$g=\frac{\sum_{i=1}^{a}\sum_{j=1}^{b}h_{i,j}}{s}$$

where *h_i,j_* is the mean error of pixel *i,j*, *f* is the frame number of the camera, *m_i,j,f_* is the distance measured at pixel *i,j* in Frame *f*, *n* = 30, *r_i,j_* is the real distance, *a* and *b* are the row and column number of the selected region respectively and *s* is the total number of pixels. The real distance *r_i,j_* is provided by the laser scanner.

**Figure 4:**
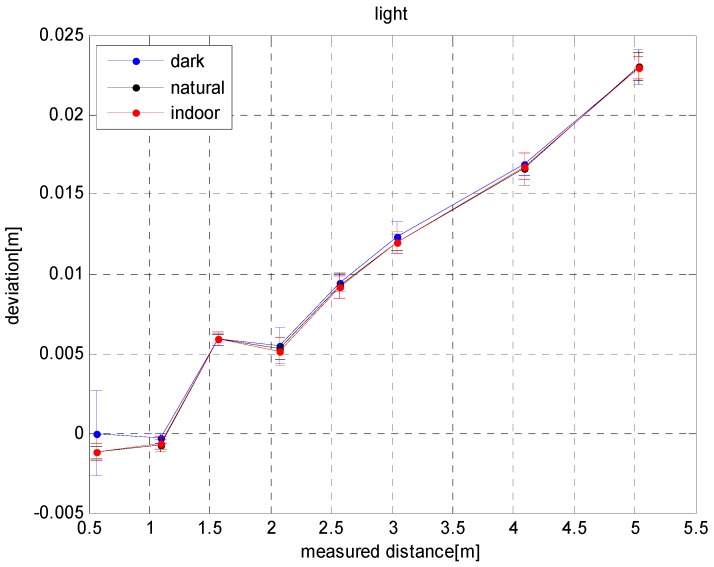
Influence of lighting on depth errors.

Figure 4 shows the effects of different lighting conditions on the depth error of the SR4000. As shown in Figure 4, the depth error of the SR4000 is on slightly affected by the lighting conditions (the maximum effect is 2 mm). The depth error increases approximately linearly with distance, and the measurement error value complies with the error test of other Swiss Ranger cameras in [37,38,39,40]. Besides, as seen in the figure, SR4000 is very robust against light changes, and can adapt to various indoor lighting conditions for the lower accuracy requirements.

**Figure 5:**
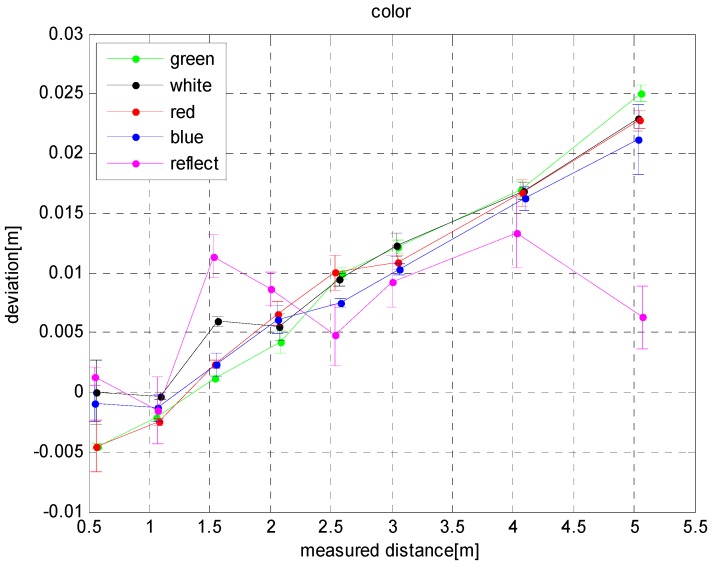
Influence of color on depth errors.

Figure 5 shows the effects of various colors on the depth errors of the SR4000 camera. As shown in Figure 5, the depth error of the SR4000 is affected by the color of the target object, and it increases linearly with distance. The depth error curve under reflective conditions is quite different from the others. When the distance is 1.5–2 m, the depth error is too large, while at 3–5 m, it is small. When the distance is 5 m, the depth error is 15 mm less than when the color is blue. When the distance is 1.5 m, the depth error when the color is white is 5 mm higher than when the color is green.

### 3.2. Influence of Material on Depth Errors

**Figure 6:**
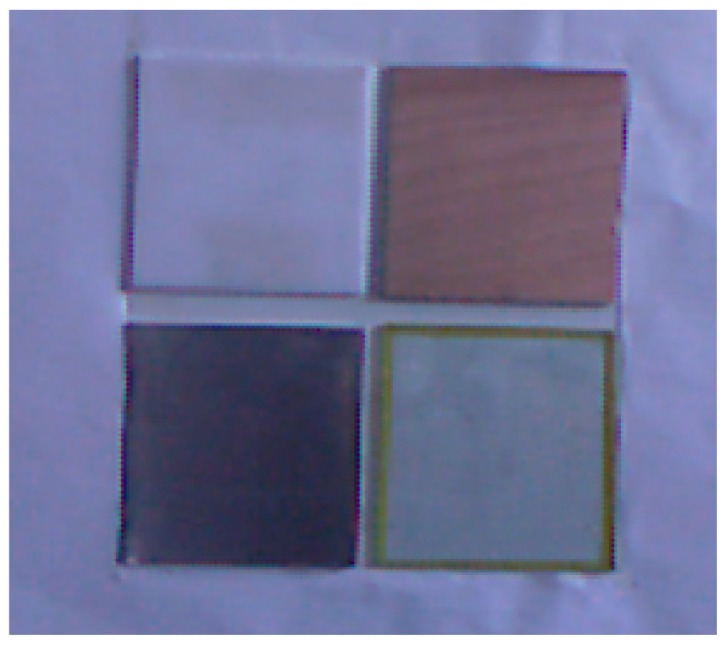
Four boards made of different materials.

During the measurement process of ToF cameras, it seems that the measured objects tend to be of different materials. Then, will this affect the measurement results? For this question, we conducted the following experiments: to analyze the effects of different materials on the depth errors of the SR4000, we chose four common materials in the experiment: ABS plastic, stainless steel, wood and glass. The tripods are arranged as shown in Figure 3 of Section 3.1, and the targets are four 5-cm-thick boards of the different materials, as shown in Figure 6. The tripods are placed parallel to the target and the distance is set to about 1 m, and the experiment is operated under natural light conditions. To differentiate the boards on the depth image, we leave a certain distance between them. Then we acquire one frame with the laser scanner and 30 consecutive frames with the SR4000 camera. The integral time in the SR_3D_View software of the SR4000 camera is set to “Auto”.

**Figure 7:**
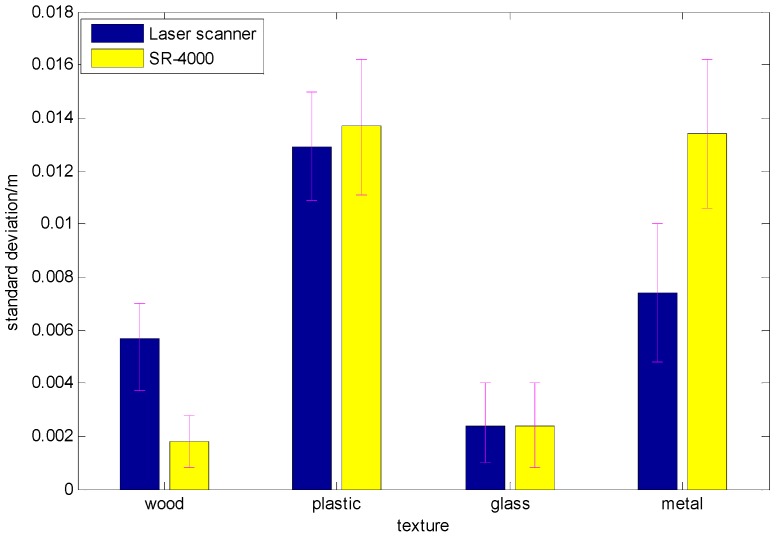
Depth data of two sensors.

For the SR4000 and the laser scanner, we select the central regions of 120 × 100 pixels and 750 × 750 pixels, respectively. To calculate the mean thickness of the four boards, we need to measure the distance between the wall and the tripods as well. Section 3.1 described the data processing method and Figure 7 shows the mean errors of the four boards.

As shown in Figure 7, the material affects both the depth errors of the SR4000 and the laser scanner. When the material is wood, the absolute error of the ToF camera is minimal and only 1.5 mm. When the target is the stainless steel board, the absolute error reaches its maximum value and the depth error is 13.4 mm, because, as the reflectivity of the target surface increases, the number of photons received by the light receiver decreases, which leads to a higher measurement error.

### 3.3. Influence of a Single Scene on Depth Errors

**Figure 8:**
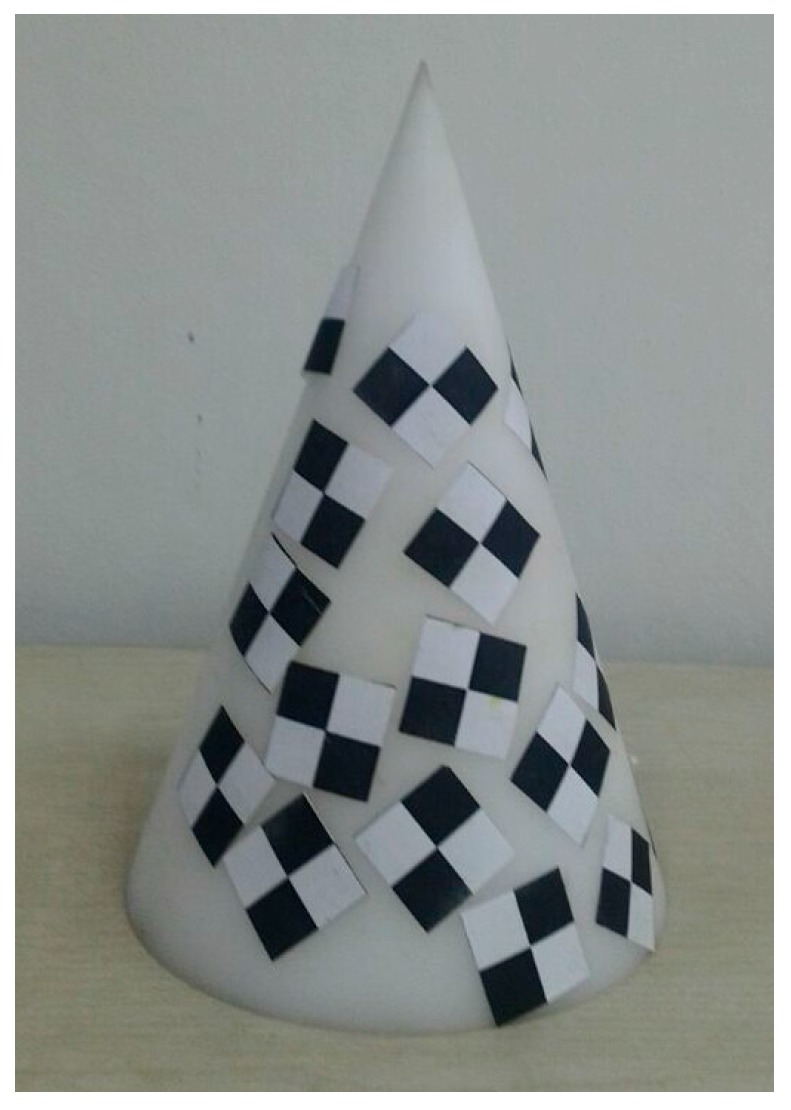
The measured cone.

The following experiments were conducted to determine the influence of a single scene on depth errors. The tripods are placed as shown in Figure 3 of Section 3.1, and as shown in Figure 8, the measuring target is a cone, 10 cm in diameter and 15 cm in height. The tripods are placed parallel to the axis of the cone and the distance is set to 1 m. The experiment is operated under natural light conditions. We acquire one frame with the laser scanner and 30 consecutive frames with the SR4000 camera. The integral time in the SR_3D_View software of the SR4000 camera is set to “Auto”.

**Figure 9:**
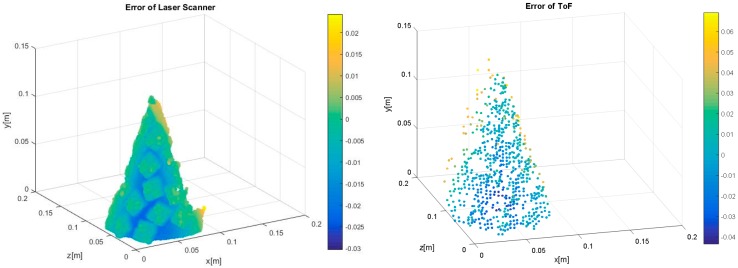
Measurement errors of the cone.

As shown in Figure 9, we choose one of the 30 consecutive frames to analyze the errors, extract point cloud data from the selected frame and compare it with the standard cone to calculate the error. The right side in Figure 9 is a color belt of the error distribution, of which the unit is m. As shown in Figure 9, the measurement accuracy of SR4000 is also higher, where the maximal depth error is 0.06 m. The depth errors of the SR4000 mainly locate in the rear profile of the cone. The measured object deformation is small, but, compared with the laser scanner, its point cloud data are sparser.

### 3.4. Influence of a Complex Scene on Depth Errors

**Figure 10:**
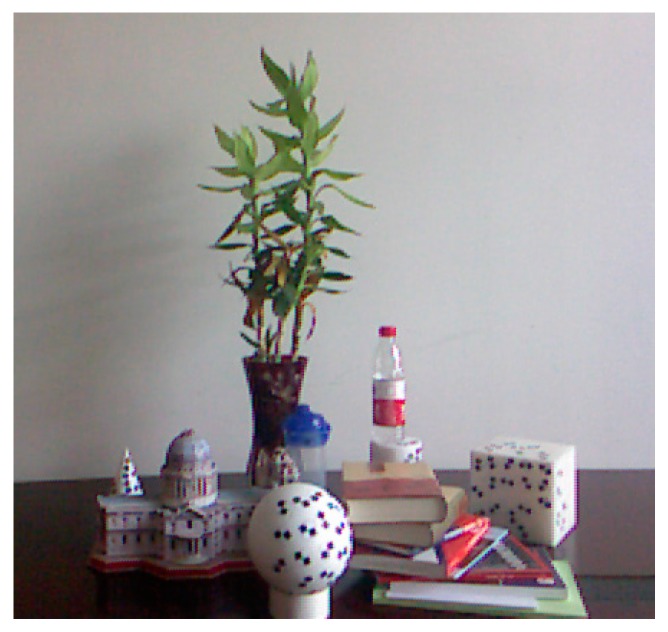
Complex scene.

The following experiments were conducted in order to determine the influence of a complex scene on depth errors. The tripods are placed as shown in Figure 3 of Section 3.1 and the measurement target is a complex scene, as shown in Figure 10. The tripods are placed parallel to the wall, and the distance is set to about 1 m. The experiment is operated under natural light conditions. We acquire one frame with the laser scanner and 30 consecutive frames with the SR4000 camera. The integral time in the SR_3D_View software of the SR4000 camera is set to “Auto”.

**Figure 11:**
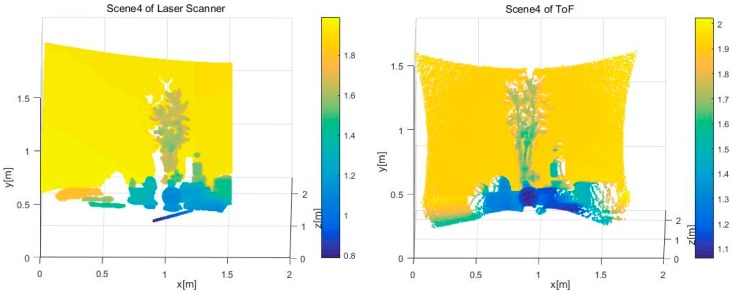
Depth images based on the point cloud of depth sensors.

We then choose one of the 30 consecutive frames for analysis and, as shown in Figure 11, obtain the point cloud data of the SR4000 and the laser scanner. As shown in Figure 11, there is a small amount of deformation in the shape of the target object measured by the SR4000 compared to the laser scanner, especially on the edge of the sensor where the measured object is clearly curved. However, distortion exists on the border of the point cloud data and artifacts appear on the plant.

### 3.5. Analysis of Depth Errors

From the above four groups of experiments, the depth errors of the SR4000 are weakly affected by lighting conditions (2 mm maximum under the same conditions). The second factor is the target object color. Under the same conditions, this affects the depth error by a maximum of 5 mm. On the other hand, the material has a great influence on the depth errors of ToF cameras. The greater the reflectivity of the measured object material, the greater the depth error, which increases approximately linearly with the distance between the measured object and ToF camera. In a more complex scene, the depth error of a ToF camera is greater. Above all, lighting, object color, material, distance and complex backgrounds could cause different influences on the depth errors of ToF cameras, but it’s difficult to summarize this in an error law, because the forms of these errors are uncertain.

## 4. Depth Error Correction for ToF Cameras

In the last section, four groups of experiments were conducted to analyze the influence of several external factors on the depth errors of ToF cameras. The results of our experiments indicate that different factors have different effects on the measurement results, and it is difficult to establish a unified model to describe and correct such errors. For a complex process that is difficult to model mechanically, an inevitable choice is to use actual measurable input and output data to model. Machine learning is proved to be an effective method to establish non-linear process models. It maps the input space to the output space through a connection model, and the model can approximate a non-linear function with any precision. SVM is a new generic learning method developed on the basis of a statistical learning theory framework. It can seek the best compromise between the complexity of the model and learning ability according to limited sample information so as to obtain the best generalization performance [41,42]. Also in the last section of this paper, we learn and model the depth errors of ToF cameras by using a LS-SVM [43] algorithm.

**Figure 12:**
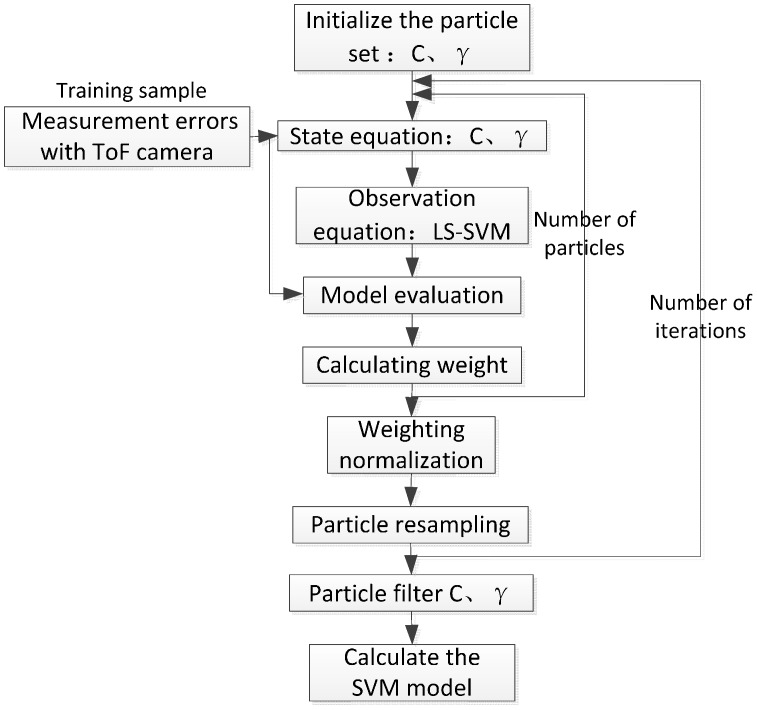
Process of PF-SVM algorithm.

Better parameters generate better SVM recognition performance to build the LS-SVM model. We need to determine the penalty parameter C and Gaussian kernel parameter γ. Cross-validation [44] is a common method which suffers from large computation demands and long running times. A particle filter [45] can be used to approximate the probability distribution of parameters in the parameter state space by spreading a large number of weighted discrete random variables, based on which, this paper puts forward a parameter selection algorithm, which can fit the depth errors of ToF cameras quickly and meet the requirements of correcting the errors. The process of the PF-SVM algorithm is shown in Figure 12 below.

### 4.1. PF-SVM Algorithm

#### 4.1.1. LS-SVM Algorithm

According to statistical theory, during the process of black-box modeling for non-linear systems, training set {*x_i_*,*y_i_*}, *i* = 1,2,…,*n* is generally given and non-linear function *f* is established to minimize Equation (8):

(7) $$f\left(x\right)=w^{T}\varphi \left(x\right)+b,$$

(8) $$\min_{w,b,\delta }J\left(w,\delta \right)=\frac{1}{2}w^{T}w+\frac{1}{2}C\sum_{i=1}^{n}\delta^{2},$$

where *ϕ*(*x*) is a nonlinear function, and *w* is the weight. Moreover, Equation (8) satisfies the constraint:

(9) $$y_{i}=w^{T}\varphi \left(x_{i}\right)+b+\delta_{i},i=1,2\cdots n,$$

where *δ_i_* ≥ 0 is the relaxation factor, and *C* > 0 is the penalty parameter.

The following equation introduces the Lagrange function *L* to solve the optimization problem in Equation (8):

(10) $$L=\frac{1}{2}{\left\|w\right\|}^{2}+\frac{1}{2}C\sum_{i=1}^{n}\delta_{i}^{2}-\sum_{i=1}^{n}\alpha_{i}\left(\varphi \left(x_{i}\right)\cdot w+b+\delta_{i}-y_{i}\right),$$

where *α_i_* is a Lagrange multiplier.

For *i* = 1,2,…*n* by elimination of *w* and *δ*, a linear equation can be obtained:

(11) $${\left[\begin{array}{cc} 0 & e^{T}\\ e & GG^{T}+C^{-1}I \end{array}\right]}_{\left(n+1\right)\times \left(n+1\right)}\left[\begin{array}{c} b\\ \alpha \end{array}\right]=\left[\begin{array}{c} 0\\ y \end{array}\right],$$

where *e* is an element of one *n*-dimensional column vector, and *I* is the *n* × *n* unit matrix:

(12) $$G={\left[\begin{array}{cccc} \varphi {\left(x_{1}\right)}^{T} & \varphi {\left(x_{2}\right)}^{T} & \cdots & \varphi {\left(x_{n}\right)}^{T} \end{array}\right]}^{T},$$

According to the Mercer conditions, the kernel function is defined as follows:

(13) $$K\left(x_{i},x_{j}\right)=\varphi \left(x_{i}\right)\cdot \varphi \left(x_{j}\right),$$

We substitute Equations (12) and (13) into Equation (11) to get a linear equation from which *α* and *b* can be determined by the least squares method. Then we can obtain the non-linear function approximation of the training data set:

(14) $$y\left(x\right)=\sum_{i=1}^{n}\alpha_{i}K\left(x,x_{i}\right)+b,$$

#### 4.1.2. PF-SVM Algorithm

The depth errors of ToF cameras mentioned above are used as training sample sets {*x_i,_y_i_*}, *i* = 1,2,…*n*, where *x_i_* is the camera measurement distance, and *y_i_* is the camera measurement error. Then the error correction becomes a black-box modeling problem of a nonlinear system. Our goal is to determine the nonlinear model *f* and correct the measurement error with it.

The error model of ToF cameras obtained via the LS-SVM method is expressed in Equation (14). In order to seek a group of optimal parameters for the SVM model to approximate the depth errors in the training sample space, we put this model into a Particle Filter algorithm.

In this paper, the kernel function is:

(15) $$k\left(x,y\right)=\exp\left(\frac{-{\left\|x-y\right\|}^{2}}{2\gamma^{2}}\right),$$

(1) Estimation state.

The estimated parameter state *x* at time *k* is represented as:

(16) $$x_{0}^{j}={\left[\begin{array}{cc} C_{0}^{j} & \gamma_{0}^{j} \end{array}\right]}^{T},$$

where $x_{0}^{j}$ is *j*-th particle when *k* = 0, *C* is the penalty parameter and *γ* is the Gauss kernel parameter.

(2) Estimation Model.

The relationship between parameter state *x* and parameter *α,b* in non-linear model *y*(*x*) can be expressed by state equation *z*(*α,b*):

(17) z(α,b)=F(γ,C),

(18) [bα1⋮αn]=[01⋯11K(x1,x1)⋯K(x1,xn)⋮⋮⋱⋮1K(xn,x1)⋯K(xn,xn)+1C]−1[0y1⋮yn],

where Equation (17) is the deformation of Equation (11).

The relationship between parameter *α,b* and ToF camera error *y*(*x*) can be expressed by observation equation *f*:

(19) y(x)=f(α,b),

(20) $$y\left(x\right)=\sum_{i=1}^{n}\alpha_{i}K\left(x,x_{i}\right)+b,$$

where Equation (20) is the non-linear model derived from LS-SVM algorithm.

(3) Description of observation target.

In this paper, we use *y_i_* of training set {*x_i,_y_i_*} as the real description of the observation target, namely the real value of the observation:

(21) $$z=\left\{y_{i}\right\},$$

(4) The calculation of the characteristic and the weight of the particle observation.

This process is executed when each particle is under characteristic observation. Hence the error values of the ToF camera are calculated according to the sampling of each particle in the parameter state *x*:

(22) $${\bar{z}}^{j}\left({\bar{\alpha }}^{j},{\bar{b}}^{j}\right)=F\left(\gamma^{j},C^{j}\right),$$

(23) $${\bar{y}}^{j}\left(x\right)=f\left({\bar{\alpha }}^{j},{\bar{b}}^{j}\right),$$

Here we compute the similarity between the ToF camera error values and the observed target camera values of each particle. The similarity evaluation *RMS* is defined as follows:

(24) $$RMS=\sqrt{\frac{1}{n}{\sum_{i=1}^{n}\left({\bar{y}}^{j}-y_{i}\right)}^{2}},$$

where ${\bar{y}}^{j}$ is the observation value of particle *j* and $y_{i}$ is the real error value. The weight value of each particle is calculated according to the Equation (24):

(25) $$w\left(j\right)=\frac{1}{\sqrt{2\pi \sigma }}e^{-\frac{RMS^{2}}{2\sigma }},$$

Then the weight values are normalized:

(26) $$w^{j}=\frac{w^{j}}{\sum_{j=1}^{m}w^{j}},$$

(5) Resampling

Resampling of the particles is conducted according to the normalized weights. In this process, not only the particles with great weights but also a small part of particles with small weights should be kept down.

(6) Outputting particle set $x_{0}^{j}={\left[\begin{array}{cc} C_{0}^{j} & \gamma_{0}^{j} \end{array}\right]}^{T}$. This particle set is the optimal LS-SVM parameter.

(7) The measurement error model of ToF cameras can be obtained by introducing the parameter into the LS-SVM model.

### 4.2. Experimental Results

We’ve performed three groups of experiments to verify the effectiveness of the algorithm. In Experiment 1, the depth error model of ToF cameras was modeled with the experimental data in [32], and the results were compared with the error correction results in the original text. In Experiment 2, the depth error model of ToF cameras was modeled with the data in Section 3.1, and the error correction results under different test conditions were compared. In Experiment 3, the error correction results under different reflectivity and different texture conditions were compared.

#### 4.2.1. Experiment 1

**Figure 13:**
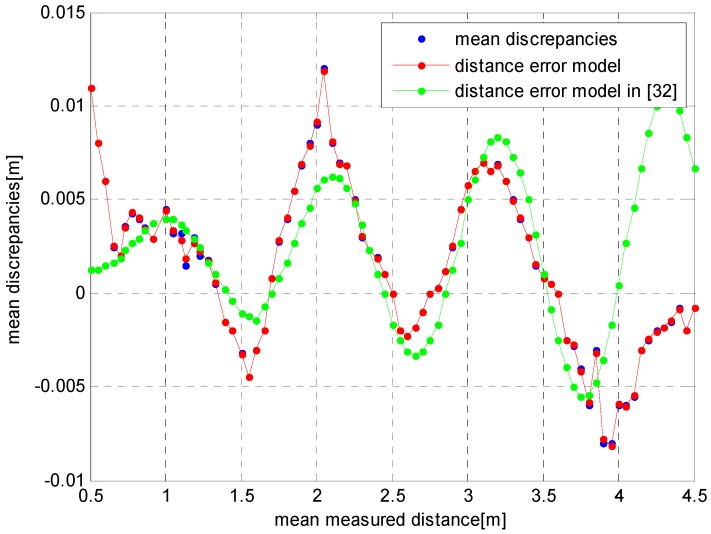
Depth error and error model.

In this experiment, we used the depth error data of the ToF cameras which was obtained from Section 3.2 of [32] as the training sample set. The training set consists of 81 sets of data, where x is the distance measurement of the ToF camera and y is the depth error of the ToF camera, as shown in Figure 13 by blue dots. In the figure, the solid green line represents the error modeling results by using the polynomial given in [32]. It shows that the fitting effect is better when the distance is 1.5–4 m, and the maximum absolute error is 8 mm. However, when the distance is less than 1.5 m or more than 4 m, the error model deviated from the true error values. By using our algorithm, we can obtain the results *C* = 736 and *γ* = 0.003. By substituting these two parameters into the abovementioned algorithm, we can also obtain the depth error model of the ToF camera as shown in the figure by the red solid line. For this, it can be seen that the error model can match the real errors well.

**Figure 14:**
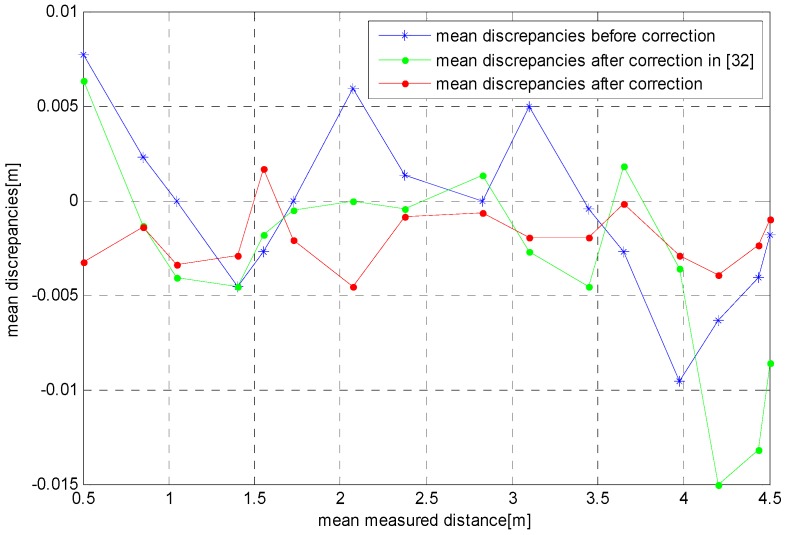
Depth error correction results.

In order to verify the validity of the error model, we use the ToF camera depth error data obtained from Section 3.3 of [32] as a test sample set (the measurement conditions are the same as Section 3.2 of [32]). The test sample set consists of 16 sets of data, as shown in Figure 14 by the blue line. In the figure, the solid green line represents the error modeling results by using the polynomial in [32]. It shows that the fitting effect is better when the distance is 1.5–4 m, and the maximum absolute error is 8.6 mm. However, when the distance is less than 1.5 m or more than 4 m, the error model has deviated from the true error value. The results agree with the fitting effect of the aforementioned error model. The model correction results obtained by using our algorithm are shown by the red solid line in the figure. It shows that the results of the error correction are better when the distance is in 0.5–4.5 m, and the absolute maximum error is 4.6 mm. Table 2 gives the detailed performance comparison results of these two error corrections. From Table 2, we can see that, while expanding the range of error correction, this method can also improve the accuracy of the error correction.

#### 4.2.2. Experiment 2

**Figure 15:**
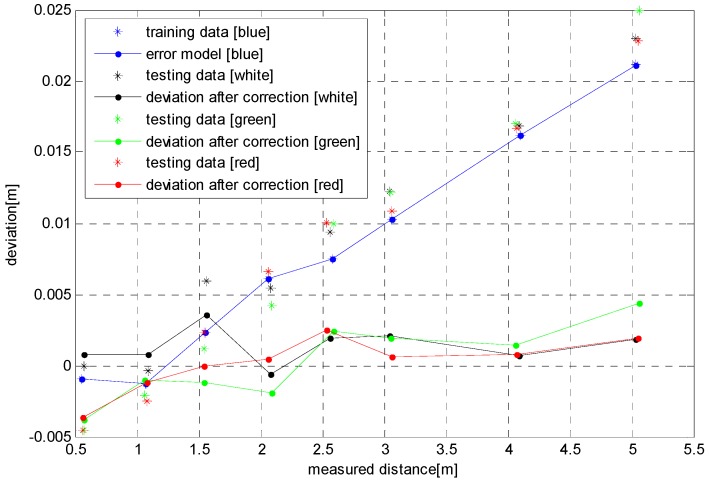
Depth error correction results of various colors and error model.

The ToF depth error data of Section 3.1 on the condition of blue background is selected as the training sample set. As shown in Figure 15 by blue asterisks, the training set consists of eight sets of data. The error model established by our algorithm is shown by the blue line in Figure 15. The model can fit the error data well, but the training sample set should be as rich as possible in order to build the accuracy of the model. To verify the applicability of the error model, we use white, green and red background ToF depth error data as test samples, and the data after correction is shown in the figure by the black, green and red lines. It can be seen from the figure that the absolute values of the three groups of residual errors is less than the uncorrected error data after the application of the blue distance error model. The figure also illustrates that this error model is very applicable to the error correction of ToF cameras for different color backgrounds.

#### 4.2.3. Experiment 3

**Figure 16:**
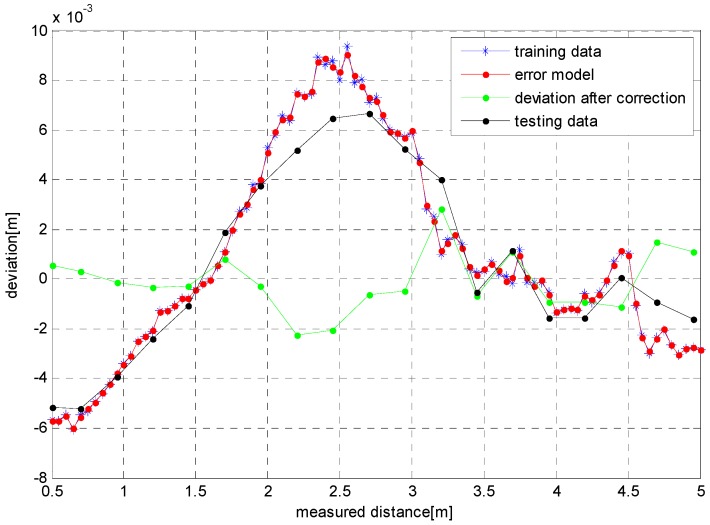
Depth error correction results and error model.

The experimental process similar to that of Section 3.1 hereof was adopted in order to verify the validity of the error modeling method under different reflectivity and different texture conditions. The sample set, including 91 groups of data, involved the depth errors obtained from the white wall surfaces photographed with a ToF camera at different distances, as shown with the blue solid lines in Figure 16. The error model established by use of the algorithm herein is shown with the red solid lines in Figure 16. The figure indicates that this model fits the error data better. With a newspaper fixed on the wall as the test target, the depth errors obtained with a ToF camera at different distances are taken as the test data, as shown with the black solid lines in Figure 16, while the data corrected through the error model created here are shown with the green solid lines in the same figure. It can be seen from the figure that the absolute values of residual errors is less than the uncorrected error data after the application of the distance error model. The figure also illustrates that this error model is very applicable to the full measurement range of ToF cameras.

## 5. Conclusions

In this paper, we analyzed the influence of some typical external distractions, such as material properties and color of the target object, distance, lighting and so on on the depth errors of ToF cameras. Our experiments indicate that lighting, color, material and distance could cause different influences on the depth errors of ToF cameras. As the distance becomes longer, the depth errors of ToF cameras increase roughly linearly. To further improve the measurement accuracy of ToF cameras, this paper puts forward an error correction method based on Particle Filter-Support Vector Machine (PF-SVM). Then, the best parameters with particle filter algorithm on the basis of learning the depth errors of ToF cameras are selected. The experimental results indicate that this method can reduce the depth error from 8.6 mm to 4.6 mm within its full measurement range (0.5–5 m).

## Figures and Tables

**Table 1:** Parameters of typical commercial ToF cameras.

ToF Camera		Maximum Resolution of Depth Images	Maximum Frame Rate/fps	Measurement Rage/m	Field of View/°	Accuracy	Weight/g	Power/W (Typical/Maximum)
MESA-SR4000	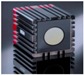	176 × 144	50	0.1–5	69 × 55	±1 cm	470	9.6/24
Microsoft-Kinect II	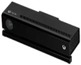	512 × 424	30	0.5–4.5	70 × 60	±3 cm@2 m	550	16/32
PMD-Camcube 3.0	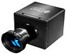	200 × 200	15	0.3–7.5	40 × 40	±3 mm@4 m	1438	-

**Table 2:** Analysis of depth error correction results.

Comparison Items	Maximal Error/mm		Average Error/mm		Variance/mm		Optimal Range/m	Running Time/s
	1.5–4	0.5–4.5	1.5–4	0.5–4.5	1.5–4	0.5–4.5		
This paper’s algorithm	4.6	4.6	1.99	2.19	2.92	2.4518	0.5–4.5	2
Reference [32] algorithm	4.6	8.6	2.14	4.375	5.34	29.414	1.5–4	-
